# Genome-Wide Divergence of DNA Methylation Marks in Cerebral and Cerebellar Cortices

**DOI:** 10.1371/journal.pone.0011357

**Published:** 2010-06-28

**Authors:** Yurong Xin, Benjamin Chanrion, Meng-Min Liu, Hanga Galfalvy, Ramiro Costa, Boro Ilievski, Gorazd Rosoklija, Victoria Arango, Andrew J. Dwork, J. John Mann, Benjamin Tycko, Fatemeh Haghighi

**Affiliations:** 1 Department of Psychiatry, Columbia University and The New York State Psychiatric Institute, New York, New York, United States of America; 2 Department of Pathology and Cell Biology, Columbia University College of Physicians and Surgeons, New York, New York, United States of America; 3 Taub Institute for Research on the Aging Brain, Columbia University College of Physicians and Surgeons, New York, New York, United States of America; 4 School Of Medicine, University Ss. Cyril and Methodius, Skopje, Macedonia; 5 Macedonian Academy of Sciences and Arts, University Ss. Cyril and Methodius, Skopje, Macedonia; RIKEN Brain Science Institution, Japan

## Abstract

**Background:**

Emerging evidence suggests that DNA methylation plays an expansive role in the central nervous system (CNS). Large-scale whole genome DNA methylation profiling of the normal human brain offers tremendous potential in understanding the role of DNA methylation in brain development and function.

**Methodology/Significant Findings:**

Using methylation-sensitive SNP chip analysis (MSNP), we performed whole genome DNA methylation profiling of the prefrontal, occipital, and temporal regions of cerebral cortex, as well as cerebellum. These data provide an unbiased representation of CpG sites comprising 377,509 CpG dinucleotides within both the genic and intergenic euchromatic region of the genome. Our large-scale genome DNA methylation profiling reveals that the prefrontal, occipital, and temporal regions of the cerebral cortex compared to cerebellum have markedly different DNA methylation signatures, with the cerebral cortex being hypermethylated and cerebellum being hypomethylated. Such differences were observed in distinct genomic regions, including genes involved in CNS function. The MSNP data were validated for a subset of these genes, by performing bisulfite cloning and sequencing and confirming that prefrontal, occipital, and temporal cortices are significantly more methylated as compared to the cerebellum.

**Conclusions:**

These findings are consistent with known developmental differences in nucleosome repeat lengths in cerebral and cerebellar cortices, with cerebrum exhibiting shorter repeat lengths than cerebellum. Our observed differences in DNA methylation profiles in these regions underscores the potential role of DNA methylation in chromatin structure and organization in CNS, reflecting functional specialization within cortical regions.

## Introduction

5-methylcytosine (5mC), a minor base in mammalian DNA, constitutes approximately 1% of DNA bases in the human genome, predominately as symmetrical methylation of the dinucleotide CpG [1]. The majority of methylated CpG dinucleotides are found within repetitive DNA elements, suggesting that cytosine methylation evolved as a mechanism for defense against transposons and other parasitic elements [2]. DNA methylation patterns change dynamically during development and are essential for X-inactivation and monoallelic expression of imprinted genes [3]. In somatic cells, promoter DNA methylation is correlated with repression of gene expression, such that CpG methylation may directly interfere with the binding of certain transcriptional factors to their cognate DNA sequences or may facilitate recruitment of methyl-CpG binding proteins that confer a repressed chromatin state [4].

DNA methylation varies among different tissues, revealing unique tissue-specific profiles. In the CNS, different DNA methylation patterns have been observed across distinct brain regions that included cerebrum, cerebellum, and pons [5]. Although this study was limited to 1,505 CpG sites confined within a panel of 807 cancer related genes, the reported differences appeared to be brain region specific and were not readily attributed to inter-individual differences in DNA methylation signatures [5], [6]. The present study significantly extends the initial efforts of Ladd-Acosta et al. (2007) [5] by (1) performing a large-scale genomic study with 377,509 CpG sites, representing over 1.3% of CpG sites in the human genome, (2) providing an unbiased genomic representation of CpG sites encompassing both intergenic and genic regions, with 170,189 CpGs represented in 16,800 known genes, and (3) determining the extent of DNA methylation in detail within prefrontal, occipital, and temporal cerebral cortex and cerebellum. These data represent the first detailed investigation of the extent of DNA methylation signatures in cerebral as compared to cerebellar cortices.

Such a large-scale genomic investigation is costly and time-consuming; hence we selected a small panel of well-characterized brain samples for DNA methylation profiling. The subjects were selected from our postmortem human brain collection with complete neuropathological and psychological data, as well as brain toxicology reports, all confirming the absence of neuropsychiatric disorders, pathological lesions, and psychoactive substances [7]. DNA methylation patterns were examined within these normal brains for four distinct regions, including prefrontal cortex, the occipital and temporal poles, and cerebellum. We used methylation-sensitive SNP chip analysis (MSNP) [8] to assess methylation patterns of these four brain regions. Our data reflects divergent DNA methylation profiles in the three regions of the cortex compared to the cerebellum, with cortex being significantly more methylated than cerebellum. These differences at selected loci reveal the potential role of DNA methylation in functional specialization of the brain.

## Results

We interrogated the DNA methylation status of 51,493 probes throughout the human genome, covering 377,509 CpG dinucleotides, with the 250K-NspI Affymetrix chip. A total of 97 250K-NspI chips were analyzed with at least two biological replicates for each sample and experimental condition. In addition to the four brain regions (i.e., prefrontal, occipital, temporal cerebral cortex and cerebellum), we also examined DNA methylation profiles within independent samples of human placenta, testes, and peripheral blood to determine whether the observed DNA methylation patterns were brain-specific. Unsupervised hierarchical clustering of the methylation (MI) scores revealed that the samples cluster by tissue and brain region (Figure S1). This tissue specificity in DNA methylation profiles is consistent with previous findings [9], [10]. Of note, is the striking hypomethylation of the placenta and cerebellum relative to other tissues and brain regions examined–though with distinct representation of hypomethylated probes in the two tissues.

**Figure 1:**
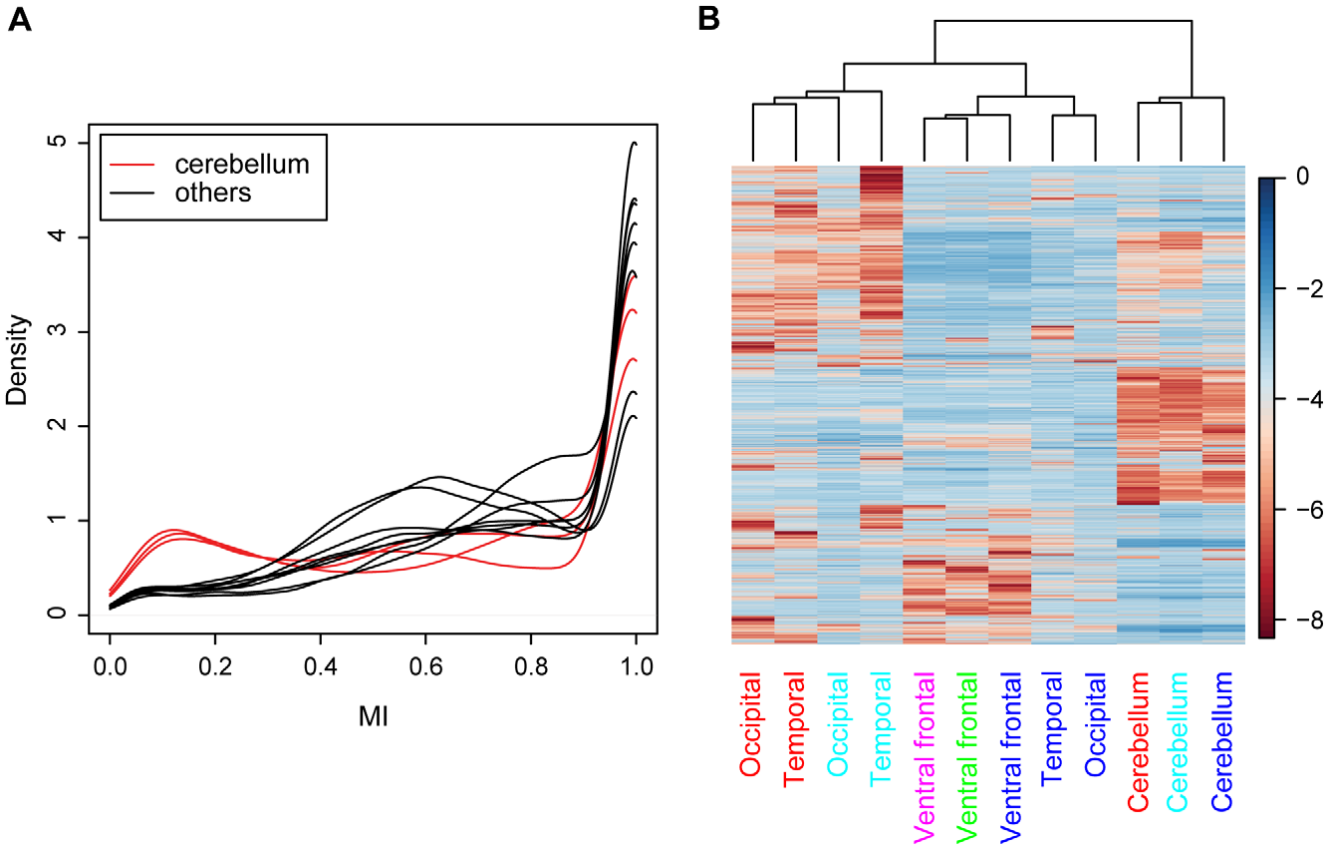
Methylation pattern of 12,180 probes. (A) Brain MI score distributions showing cerebellum samples with bimodal distributions in red and other brain regions in black. (B) Hierarchical clustering of methylation data. The heat map shows relative methylation differences depicted in (red) hypomethylated and (blue) hypermethylated log_2_(MI) scores (rows and columns corresponding to probes and samples, respectively). Dendrogram depicts clustering of samples by brain regions, where regions from the same subjects are color-coded.

Further, the brain-specific methylation signatures revealed that cerebellum is hypomethylated compared with prefrontal, occipital, and temporal regions of the cortex (Figure 1). The distribution of the MI scores shows a striking hypomethylated peak for the cerebellum samples, whereas the samples from the cortex show a shift in the corresponding peak in the hypermethylated direction (Figure 1A). Overall, the three regions of the cortex grouped together. The occipital and temporal regions of the brain clustered by subject rather than by region (Figure 1B). Such inter-individual methylation variability is perhaps not surprising, since the occipital and temporal sensory regions of the brain are subject to experience-based plasticity [11], [12]. Moreover, 41% of the probes covering 170,189 CpG sites, are located in genic regions with 97% of CpGs in gene bodies and 4.3% in promoter regions. Although the aim of this study was to examine the extent of DNA methylation genome-wide, we also examined the extent of DNA methylation within these genic regions to investigate if there are differences in DNA methylation patterns between genic and intergenic regions. Cluster analysis of genic probes also showed the same clustering pattern, with cerebellum hypomethylated (supplementary Figure S2).

To determine which probes significantly differentiate cerebral and cerebellar cortices, we used a linear mixed-effects model to identify 9,661 such probes with p≤0.001 corrected for multiple testing (supplementary Figure S3 and Table S2). Unsupervised cluster analysis of these differentially methylated probes still showed striking hypomethylation within cerebellum. The distribution of the MI scores also still retained the hypomethylated peak for the cerebellum samples, and as expected the methylation differences between the cerebellum and prefrontal, occipital, and temporal cortical samples became far more sharply defined after this analysis (Figure S3A). The majority of these probes were hypomethylated in cerebellum as compared to the three cortical regions. We next identified those probes that were significantly hypomethylated in cerebellum relative to prefrontal, occipital, and temporal regions of the cortex. A total of 1,574 significant probes, including 788 genic probes, were found with p≤0.001 corrected for multiple testing (Table S3). In contrast, only 147 probes exhibit the opposite trend, with cerebellum hypermethylated as compared with the three cortical regions.

**Figure 2:**
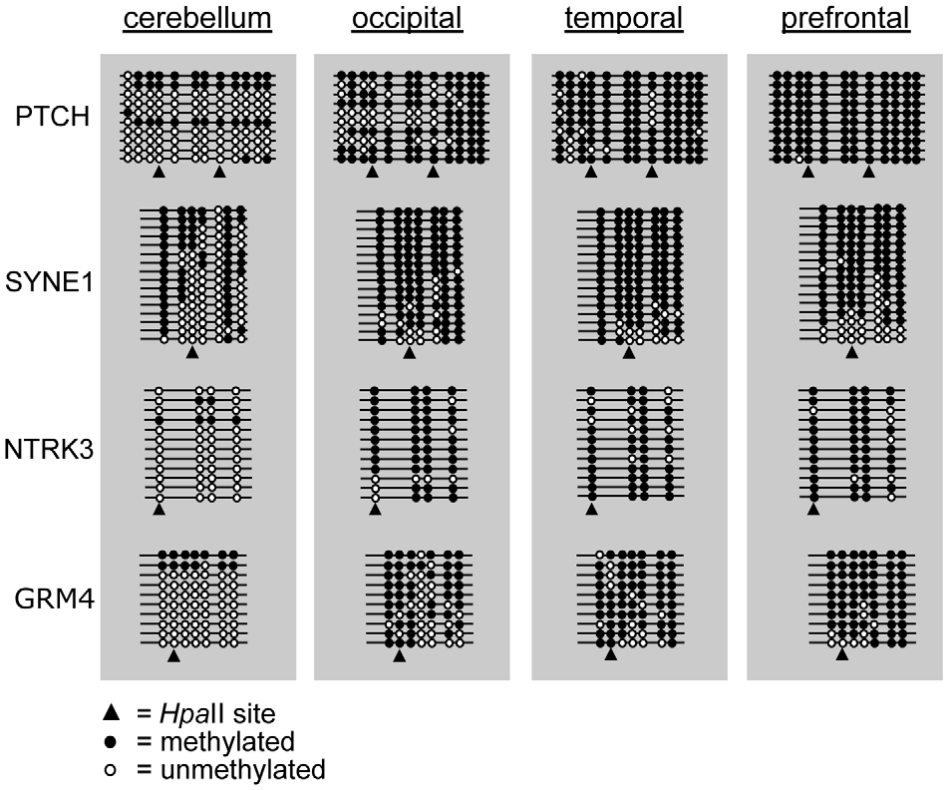
Regional validation. Regional validation via bisulfite cloning and sequencing where the cerebellum is hypomethylated vs. the cerebrum hypermethylated. Triangles identify CpGs within the index *Hpa*II sites probed by the MSNP assay, where black/white circles are methylated/unmethylated CpGs, respectively.

Examination of the genic probes identified 113 genes with distinct CNS function, determined by biological theme discovery using Gene Ontology (GO) terms (supplementary Table S4). These genes appear to be involved in diverse CNS functions, including nervous system development, transmission of nerve impulses, synaptic transmission, and neuron differentiation. Importantly, we were able to independently validate the microarray data by performing bisulfite cloning and sequencing for four of these genes, randomly selected for validation. These include *NTRK3* (neurotrophic tyrosine kinase, receptor, type 3), *PTCH1* (patched homolog 1 (*Drosophila*)), *SYNE1* (spectrin repeat containing, nuclear envelope 1), and *GRM4* (glutamate receptor, metabotropic 4) within the four brain regions examined (see Supplementary Materials S1 for experimental details). The bisulfite sequencing results for these regions consistently validate our predictions from the MSNP analysis and augment these data by demonstrating coordinate involvement of multiple CpG dinucleotides near the index *Hpa*II sites, showing that cerebellar cortex is hypomethylated as compared to three distinct regions of the cerebral cortex (Figure 2). Though a minor concern, we nevertheless conservatively performed the bisulfite cloning and sequencing validation experiments using brain regions from the same individuals to avoid potential confounds due to inter-individual variability in DNA methylation signatures. Interestingly, these genes, and others that we have identified in this study, show cerebellum-specific activity (see supplementary Figure S4 for description of genes and their function in cerebellum). These data reflect relative differences in DNA methylation levels, which may not directly influence gene expression within the tissues examined. These results reveal that DNA methylation patterns vary markedly between cerebral and cerebellar cortices and further demonstrate that the genome of the cerebellum is hypomethylated as compared to regions of the cerebral cortex, suggesting a potential role for DNA methylation in brain organization and function.

## Discussion

The extent of DNA methylation varies widely across the genome and different cells within the nervous system. Although due to experimental limitations in the requisite amount of starting DNA we did not investigate the DNA methylation profiles within individual cell populations, it is possible that some of our findings may be attributed to cellular heterogeneity in cortex and cerebellum. Additionally, the inter-individual variability within occipital and temporal cortices was larger than the tissue variability. This may be due to technical limitations of the SNP chip-based approach used for DNA methylation profiling. Still, our findings are consistent with previous observations of the chromatin architecture in cerebral and cerebellar cortices. Early studies of global chromatin structure within neurons of the cerebral cortex and cerebellum revealed that the nucleosome repeat length (i.e., the spacing between adjacent nucleosomes) correlates with developmental trajectories [13], [14], [15], [16], [17]. Nucleosome repeat lengths change concomitant with terminal differentiation. In rat cortical neurons, the chromosome repeat length shortens rapidly towards the end of the second postnatal week, coinciding with neuronal maturation [15]. In contrast, cerebellar neurons exhibit short repeat lengths at late fetal and early postnatal stages and lengthen to adult levels about 30 days postnatally [15]. These studies suggest that chromatin architecture is plastic during development, but may no longer be dynamic in adulthood. Our data profile DNA methylation patterns in adult tissue, and may represent the endpoint of the developmental methylation trajectory of cerebral and cerebellar cortices. The striking differences we observe in DNA methylation in cerebellum and regions of the cerebral cortex may contribute to functionally relevant differences in chromatin architecture.

In mammalian cells, methyl-CpGs throughout the genome are recognized by methyl binding proteins. Methyl-CpG binding proteins represent an important class of chromosomal proteins that associate with multiple protein partners to modify surrounding chromatin and to silence transcription, providing a functional link between DNA methylation and chromatin remodeling and modification. The molecular function of these proteins depends on their ability to recognize and bind methylated DNA in a site-specific manner. Bound methyl-CpG binding proteins establish silent chromatin states by forming co-repressor complexes with histone deacetylases and methylases to confer chromatin compaction, leading to transcriptional repression [4], [18].

Examination of our genome-wide methylation data in the context of previously published gene expression data in the prefrontal cortex and the cerebellum [19] suggests intriguing trends. Genes with CpG sites within promoters and gene bodies that are hypomethylated in cerebellum relative to the cortex had a bimodal distribution corresponding to a low and high pattern of gene expression (Figure S5). The proportion of highly expressed genes is greater in cerebellum, which is hypomethylated relative to the cortex. In contrast, the low expressed genes appear to be enriched in the cortex (Figure S5) as compared to the cerebellum. These trends are consistent with the expected role of promoter DNA methylation in transcriptional repression. Yet, in our data the majority of CpG sites reside within gene bodies and intergenic regions and may not be directly associated with expression of proximal gene(s). In a recent genome-wide study, highly expressed genes were found to have hypomethylated promoters and hypermethylated gene bodies [20]. Further, it has been reported that CpG methylation may influence gene expression long range, with some tissue-specific differentially methylated regions having been observed up to 3MB away from CpG islands within gene bodies and upstream transcription start sites [10]. The influence of CpG methylation within genomic regions that are not immediate to promoters or transcription start sites is still poorly understood, however methylation at these sites is likely highly critical in chromatin remodeling and thus genome organization and function.

Our findings showing divergence of DNA methylation profiles in regions of cerebral cortex and cerebellum highlight the impact of DNA methylation on higher-order chromatin structure. Such regional differences in DNA methylation were likely acquired developmentally as part of brain functional specialization, underscoring the potential role of DNA methylation in brain development and neuronal differentiation. Investigation of DNA methylation during cortical development has revealed that these patterns are dynamic, indicating methylation alterations in CpG island promoters of genes involved in CNS growth and development [21]. Taken together, these findings suggest that DNA methylation, in concert with other chromatin remodeling factors, maybe essential in mediating global morphological and/or functional changes during neuronal differentiation and development. Proper establishment and maintenance of DNA methylation marks early in neurodevelopment can mediate proper maturation of the complex neurocircuitry in cerebellum and prefrontal, occipital, and temporal cortices, influencing gene expression and ultimately brain function and behavior.

## Materials and Methods

### Ethics Statement

Genomic DNA from normal human tissue specimens with anonymized identifiers was used under exemption by the Columbia University and New York State Psychiatric Institute institutional review board.

### Samples and Subjects

We examined a total of 18 samples, consisting of 12 brain samples and six samples that include two each of placenta, testes, and peripheral blood. The non-CNS specimens were each obtained from independent subjects. The 12 brain samples studied were derived from four regions (cerebellum, occipital, temporal, and ventral frontal cortices) of 5 brains (Table S1), consisting of four males, average age 36±2 years, and one female, age 41. Brain pH ranged from 5.84 to 6.79, and the average postmortem interval (PMI) was 14±3.8 hours. Methylation patterns did not differ significantly by age, sex, pH, and PMI within specimens examined. The brains were derived from forensic autopsies. With informed consent, family members were interviewed for a psychological autopsy to obtain demographic information and any evidence of psychiatric or somatic disease. The right cerebral hemisphere was sliced coronally at intervals of 2–5 cm, and the slices were rapidly frozen in Freon (dichlorodifluoromethane or 1,1,1,2 tetrafluoroethane), as were the cerebellum and brainstem. Frozen specimens were then stored at −80°C until use. Most of the left cerebral hemisphere was fixed in phosphate-buffered formalin for diagnostic and other histological studies. For the current study, slices were warmed to no more than −20°C, and samples of ∼200–500 mg were cut with a scalpel from folia of the right superior and inferior semilunar lobules of the cerebellum, and cerebral cortex from the caudal end of the right superior occipital gyrus, the lateral aspect of the rostral end of the right superior temporal gyrus, and the rostral end of the right gyrus rectus and orbital gyrus. Samples were kept at −80°C until further processing.

### MSNP Procedure

We describe this procedure here only briefly because we have previously reported the MSNP method [8]. Genomic DNA was purified using the DNeasy Blood and Tissue Kit (Qiagen). We used the protocol of the SNP array manufacturer (Affymetrix) for the 250K *Nsp*I chip with the addition of an initial *Hpa*II or *Msp*I digestion for the plus-*Hpa*II and plus-*Msp*I probes. Genomic DNA (250 ng per representation) was digested with *Hpa*II or *Msp*I in a total volume of 12 µl (Buffer 1, New England Biolabs) for 3 hrs, followed by adjustment of the buffer by addition of 15.8 µl of water and 3.2 µl of 10× Buffer 3 (New England Biolabs), a further digestion with *Nsp*I for 3 hrs with *Nsp*I linkers ligated to the restriction fragments, and PCR was carried out for 30 cycles with linker primers. The same amount of genomic DNA was digested with *Nsp*I only followed by PCR. We carried out PCR purification using the DNA Amplification Clean-Up Kit (Clontech), and probe fragmentation (brief exposure to DNase), biotin-labeling and hybridization as described in the Affymetrix protocol. We determined intensity values by normalization and model-based expression (PM/MM difference model) followed by batch effect adjustment using dChip [22], [23]. The main determinant of the data quality is the starting material: the genomic DNA must be of high molecular weight. Call rates were in the range of 80–98% with minus-*Hpa*II (*Nsp*I-only) and plus-*Hpa*II representations, compared with lower call rates of 73–91%, as predicted, in the plus-*Msp*I, in which many of the SNPs dropped out as a result of complete digestion of internal CCGG sites. Further, we observed 91% average reliability in genotype call rates across all technical replicates with minus-*Hpa*II treatment.

For DNA methylation analysis, SNP classes for the 500K SNP chips were determined computationally. We used the March 2006 (hg18) build of the human genome and the SNP database to identify the SNPs on the 250K *Nsp*I chip. These SNPs are classified into three groups based on the presence and position of *Hpa*II sites within *Nsp*I fragments. Class I SNPs lack internal *Hpa*II sites so that they are not informative for DNA methylation but useful as internal controls. Class 2 SNPs have at least one internal (nonpolymorphic) *Hpa*II site. Class 3 SNPs are identical to those in Class 2, except these SNPs contain a polymorphic *Hpa*II site wherein the *Hpa*II recognition site is potentially created or abolished by a SNP, rendering these SNPs unreliable for use in DNA methylation analysis. Henceforth, we only used the Class 2 SNPs since they would be affected in their amplification and representation in the SNP chip probe preparation only by the methylation state of the (non-polymorphic) *Hpa*II site.

We evaluated the Class 2 SNPs experimentally by checking the intensity ratios for plus-*Msp*I to minus-*Hpa*II representations. The informative Class 2 SNPs by experiments are defined as those SNP-tagged loci that have more than 20% decrease in the average probe intensity ratios. Approximately 83% of the experimentally determined Class 2 SNPs overlap with the computationally predicted Class 2 SNPs. In this way we derived a set of empirical Class 2 SNPs that met the criteria of being Class 2 both *in silico* and in practice (a complete list of these SNPs are available by request from the authors).

### MSNP Data Analysis

The expression difference between the minus-*Hpa*II and plus-*Hpa*II genomic representations indicates the methylation status of examined SNPs. Since we had at least two technical replicates for each brain sample, we calculated methylation index (MI), defined as pairwise fold changes of signal intensity plus-*Hpa*II/minus-*Hpa*II, and then determined the median of these pairwise MIs for each probe within a sample. The estimated MI scores were not truncated in the statistical analyses. We further eliminated uninformative probes, defined as those with low coefficient of variation (CV≤10%) in the brain. For cluster analysis, we further eliminated probes with CV≤30%. Note, MI scores greater than 1.0 were adjusted to 1.0 for cluster analysis. Unsupervised hierarchical clustering was performed using the HOPACH package in the R statistical program [24] for hierarchical clustering and the correlation distance as the distance metric. For clustering analysis, MI data were log_2_ transformed to values ranging from −8 to 0 corresponding to the relative level of unmethylated and methylated states, respectively.

The difference in the methylation patterns in the neocortex and the cerebellar cortex was assessed using a linear mixed-effects model and evaluated by ANOVA. The mixed effect model accounted for potential correlation in signal intensities that may arise from having multiple brain regions from the same subject as well as having multiple technical replicates for the same brain regions. Significance levels for the methylation differences between the cerebellum and other brain regions were computed via SNP-by-SNP mixed effect models in the statistical software R (http://www.r-project.org/). The response was the fold change of intensity levels (MIs) for possible plus-*Hpa*II and minus-*Hpa*II combinations of array representations within the same sample. Brain region was the fixed factor, and subject ID was used as a random factor. P-values corresponding to the F statistic (and to the pairwise t-tests) were recorded. P-values were adjusted for multiple testing using the Benjamini-Hochberg linear step-up procedure [25].

## Supporting Information

Supplemental Materials S1 (0.07 MB DOC)

Figure S1 Hierarchical clustering of methylation data with 12,180 probes (with coefficient of variation, CV>0.3). The heat map shows relative methylation differences with hypomethylated (in red) and hypermethylated (in blue) log_2_(MI) scores. Dendrogram depicts clustering of samples by brain regions and tissues.
(3.47 MB TIF)

Figure S2 Hierarchical clustering of methylation data with methylation patterns of 5,284 genic probes with CV>0.3. Rows represent probes and columns represent samples. The heat map shows relative methylation differences with hypomethylated (in red) and hypermethylated (in blue) log_2_(MI) scores. Dendrogram depicts clustering of samples by brain regions and tissues.
(3.48 MB TIF)

Figure S3 Methylation patterns of 9,661 probes that significantly differentiate cerebral and cerebellar cortices with p≤0.001 corrected for multiple testing. (A) Brain MI scores showing distinct bimodal distributions for the cerebellum samples. (B) Hierarchical clustering of methylation patterns with hypomethylated (in red) and hypermethylated (in blue) log_2_(MI) scores (rows and columns representing probes and samples, respectively). Brain regions with similarly colored labels are from the same subjects. Dendrogram depicts clustering by brain regions, separated by two major branches for cerebellum and the regions of cerebral cortex.
(7.76 MB TIF)

Figure S4 (A) Schematic representation of the human brain depicting the DNA methylation profile of the neocortex (in blue) and the cerebellar cortex (in red), corresponding to hyper- and hypo-methylated states, respectively. The values depicted in the cartoon represent the average of the MI scores across all 113 CNS related genes identified. The values in the adjacent table correspond to MI scores for selected CNS genes with (B) describing the identified roles of these genes in cerebellum. The parietal cortex (shown in gray in the cartoon below) was not included in this study.
(10.77 MB TIF)

Figure S5 Gene expression profiles for 104 genes with CpG sites hypomethylated in cerebellum and hypermethylated in prefrontal cortex. Gene expression density distribution are plotted in log_2_ scale for expression intensity values (x-axis) with lines denoting cerebellum (in red) and cortex (in black). The expression data were downloaded from Gene Expression Omnibus public database (GEO accession: GSE6306).
(2.42 MB TIF)

Table S1 Postmortem brain subjects used in the MSNP experiments. The color coding for subjects is consistent with those used in Figures 1, S1, S2, and S3.
(0.03 MB DOC)

Table S2 Probes with significant differential DNA methylation in cerebral and cerebellar cortices. The table shows the MI scores of samples from prefrontal (vfrnt), occipital (occ), and temporal cortex (tmp), and the cerebellum (cer) as well as the genomic coordinate of SNP probes. Brain regions from the same subjects are color-coded. The genomic location of probes is categorized in genic (overlapping gene bodies) and inter-genic regions.
(3.29 MB XLS)

Table S3 Probes that appear to be significantly hypomethylated in cerebellum and hypermethylated in cerebrum. See the description of columns in Table S2.
(0.56 MB XLS)

Table S4 Genic probes with known CNS function. See the description of columns in Table S2.
(0.07 MB XLS)
