# Supplementary material for: Genome-Wide Divergence of DNA Methylation Marks in Cerebral and Cerebellar Cortices
Source: PLoS One. 2010 Jun 28;5(6):e11357. doi: 10.1371/journal.pone.0011357 (PMC2893206; doi:10.1371/journal.pone.0011357)
Supplement: Supplemental Materials S1 — (0.07 MB DOC) [file pone.0011357.s001.doc]

**SUPPLEMENTARY MATERIALS**

***Biological Theme Discovery for Detection of Genes with CNS Function****:* The significant SNPs were mapped to the latest human genome assembly (NCBI build 36) to find associated genes and promoters (considering 1Kb promoter region). The gene list was analyzed by DAVID functional annotation tool (Dennis et al., 2003), and genes were identified with associated functional terms involving CNS function. These terms included, nervous system and neuron development, transmission of nerve impulses, synaptic transmission, neurophysiological processes, neurogenesis, axonogenesis, neuron morphogenesis during differentiation, neurite morphogenesis, neuron differentiation, axon guidance, plexin/semaphorin/integrin, semaphorin/CD100 antigen, Sema, and Sema domain.

***Validation of the MSNP data by bisulfite cloning and sequencing:*** The methylation data for selected SNPs were validated by bisulfite conversion using the CpG genome DNA modification kit (Zymo research, Orange, CA) followed by PCR amplification, cloning, and sequencing. PCR was done using Platinum Blue PCR SuperMix (Invitrogen, Carlsbad, CA) and with locus-specific primers matching the bisulfite-converted sequences flanking the CpG dinucleotides to be assayed. PCR primers were selected using MethPrimer (<http://www.urogene.org/methprimer/index1.html>), with sequences provided in the supplementary table below. Primers were tested for linearity on standards with known methylation levels. Bisulfite-converted/PCR-amplified DNA was cloned using the TOPO-TA Cloning kit (Invitrogen) and at least 12 clones were sequenced.

| **PCR primers and conditions used in this study.** | | | | | |
| --- | --- | --- | --- | --- | --- |
| Gene/  Region | Index SNP | Forward Primer | Reverse Primer | Anneal. Temp (°C) | Size (bp) |
| SYNE1 | SNP_A-4229152 | AAGTAAGTTGGAGGAAAATAGATTATATAG | AAAAAACTCCTTTTTATCAAATACTC | 61.8>62.9 | 322 |
| GRM4 | SNP_A-1840874 | TGGTTTAAATGAGTAGTTGTAATTATG | TTTAAACAACCAAAAACATCTAAATTC | 60.2>61.3 | 330 |
| NTRK3 | SNP_A-1925241 | GAGAGTTAGTATTTTTGGGAAAGTT | TCATTAAATATTAACAACAAAACACAATAC | 61.7>61 | 203 |
| PTCH1 | SNP_A-1997271 | AATTTTTTGTTATTGTTGGTAGTAT | TACATAAACATCCTATTATTCATTTAC | 59>58.3 | 364 |

**References**
