# Supplementary figures and images for: Genome-Wide Divergence of DNA Methylation Marks in Cerebral and Cerebellar Cortices
Source: PLoS One. 2010 Jun 28;5(6):e11357. doi: 10.1371/journal.pone.0011357 (PMC2893206; doi:10.1371/journal.pone.0011357)

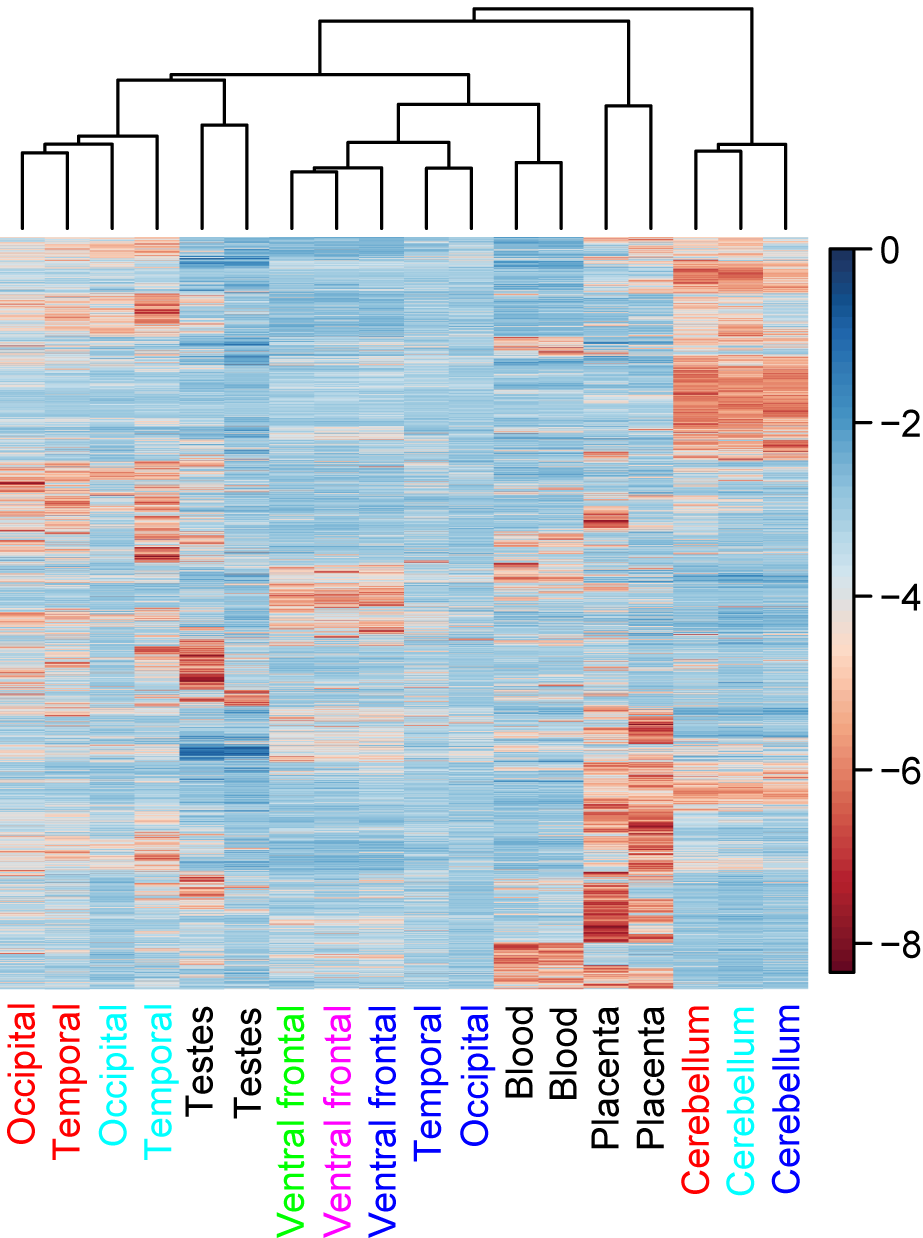

Supplement: Figure S1 — Hierarchical clustering of methylation data with 12,180 probes (with coefficient of variation, CV>0.3). The heat map shows relative methylation differences with hypomethylated (in red) and hypermethylated (in blue) log2(MI) scores. Dendrogram depicts clustering of samples by brain regions and tissues. (3.47 MB TIF) [file pone.0011357.s002.tif]

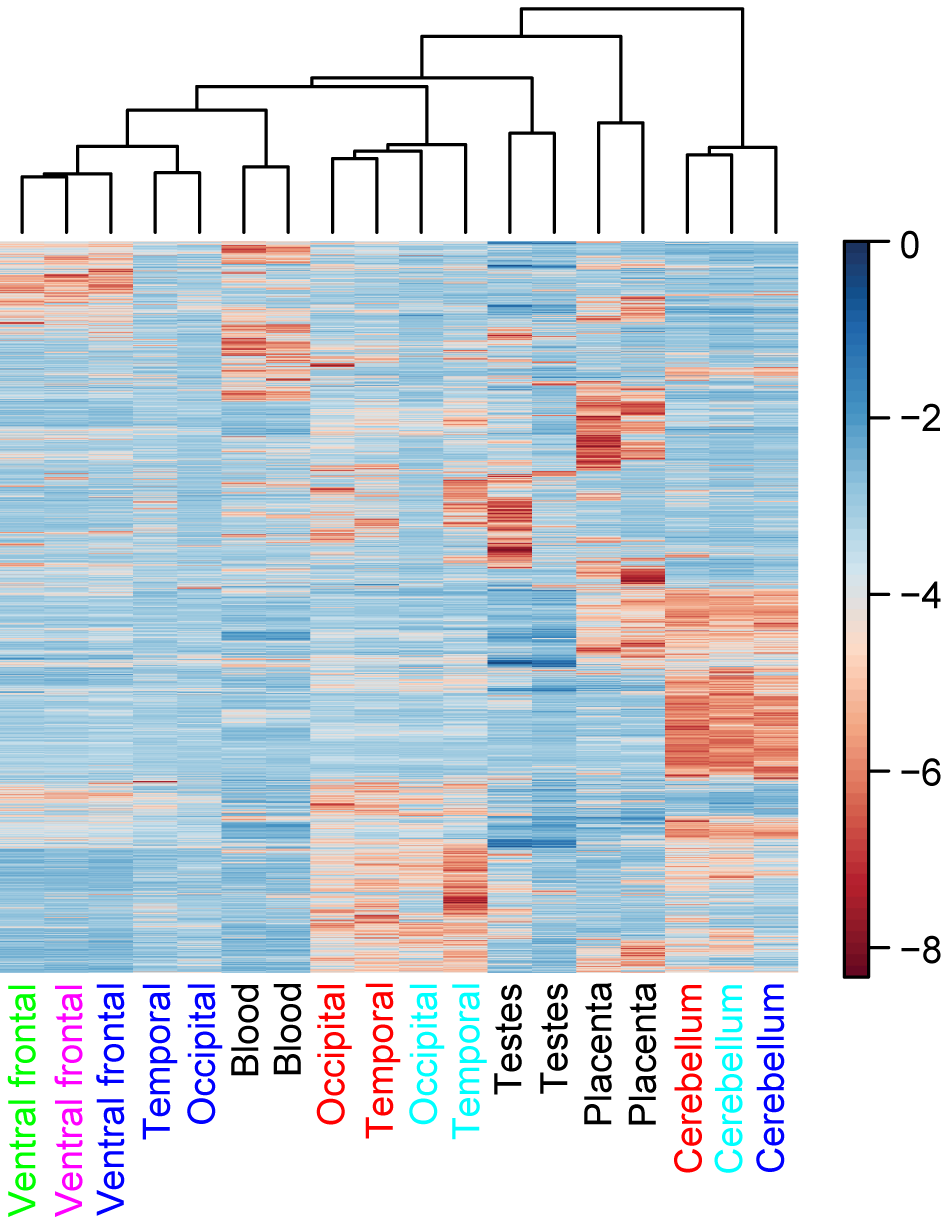

Supplement: Figure S2 — Hierarchical clustering of methylation data with methylation patterns of 5,284 genic probes with CV>0.3. Rows represent probes and columns represent samples. The heat map shows relative methylation differences with hypomethylated (in red) and hypermethylated (in blue) log2(MI) scores. Dendrogram depicts clustering of samples by brain regions and tissues. (3.48 MB TIF) [file pone.0011357.s003.tif]

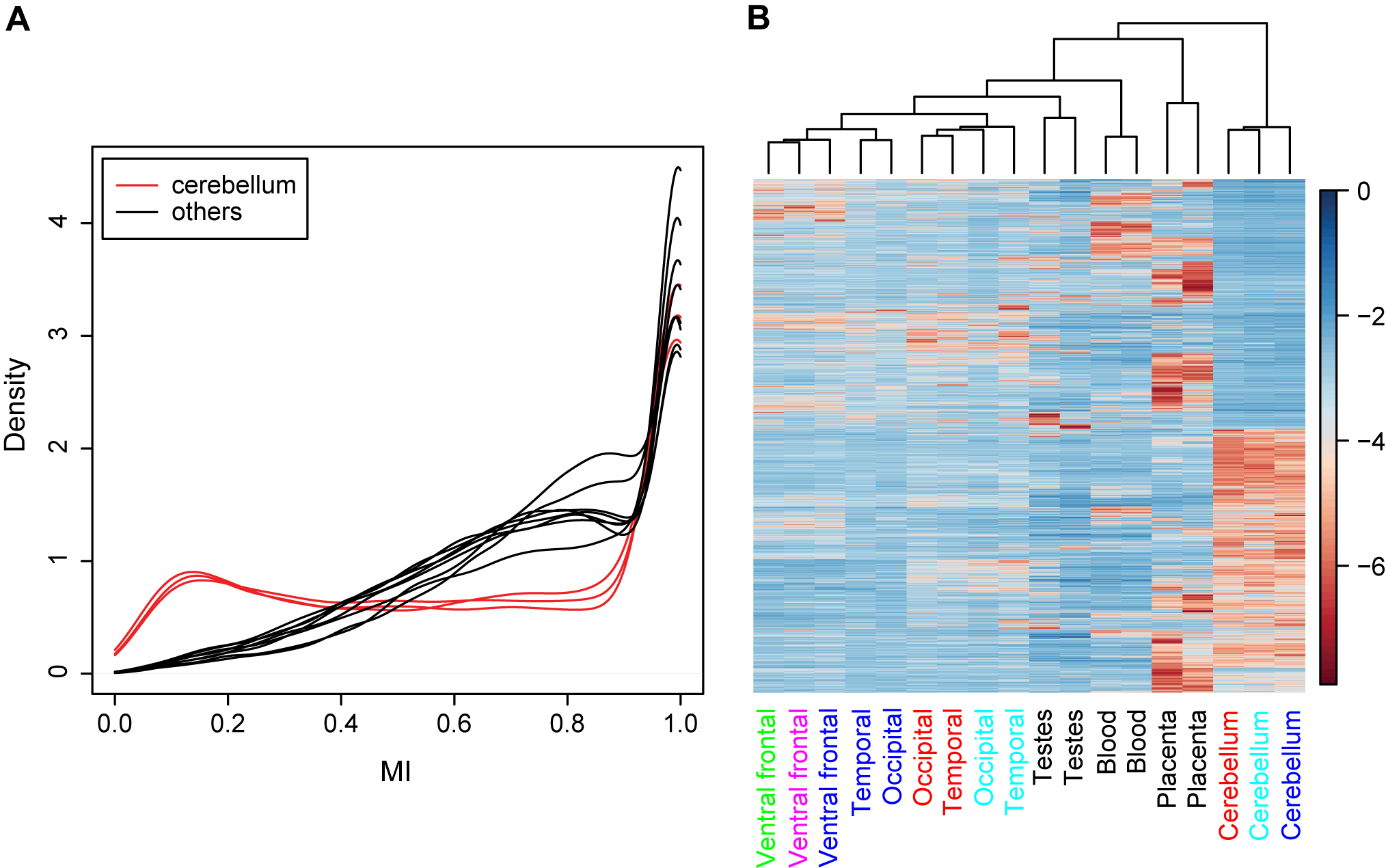

Supplement: Figure S3 — Methylation patterns of 9,661 probes that significantly differentiate cerebral and cerebellar cortices with p≤0.001 corrected for multiple testing. (A) Brain MI scores showing distinct bimodal distributions for the cerebellum samples. (B) Hierarchical clustering of methylation patterns with hypomethylated (in red) and hypermethylated (in blue) log2(MI) scores (rows and columns representing probes and samples, respectively). Brain regions with similarly colored labels are from the same subjects. Dendrogram depicts clustering by brain regions, separated by two major branches for cerebellum and the regions of cerebral cortex. (7.76 MB TIF) [file pone.0011357.s004.tif]

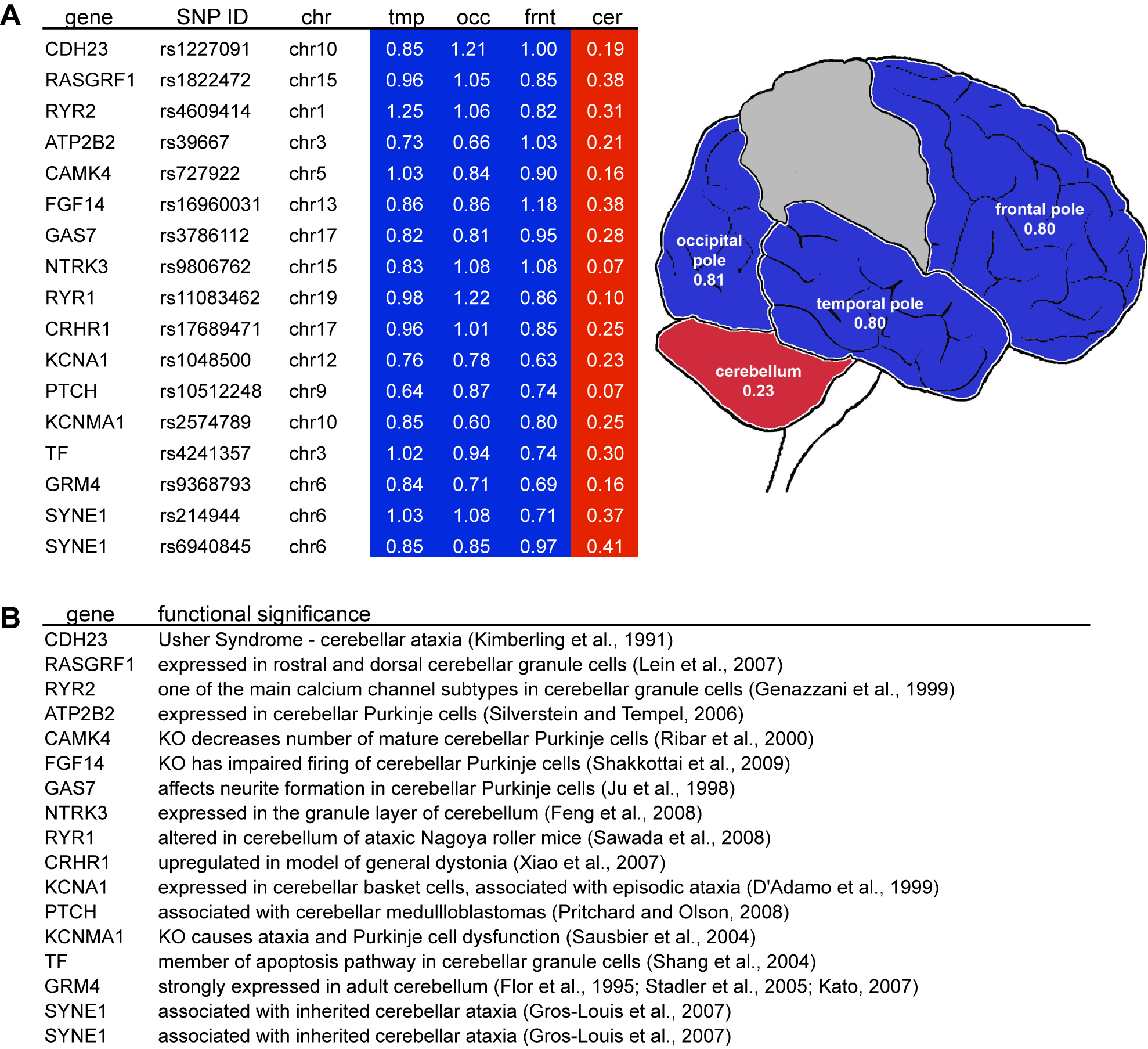

Supplement: Figure S4 — (A) Schematic representation of the human brain depicting the DNA methylation profile of the neocortex (in blue) and the cerebellar cortex (in red), corresponding to hyper- and hypo-methylated states, respectively. The values depicted in the cartoon represent the average of the MI scores across all 113 CNS related genes identified. The values in the adjacent table correspond to MI scores for selected CNS genes with (B) describing the identified roles of these genes in cerebellum. The parietal cortex (shown in gray in the cartoon below) was not included in this study. (10.77 MB TIF) [file pone.0011357.s005.tif]

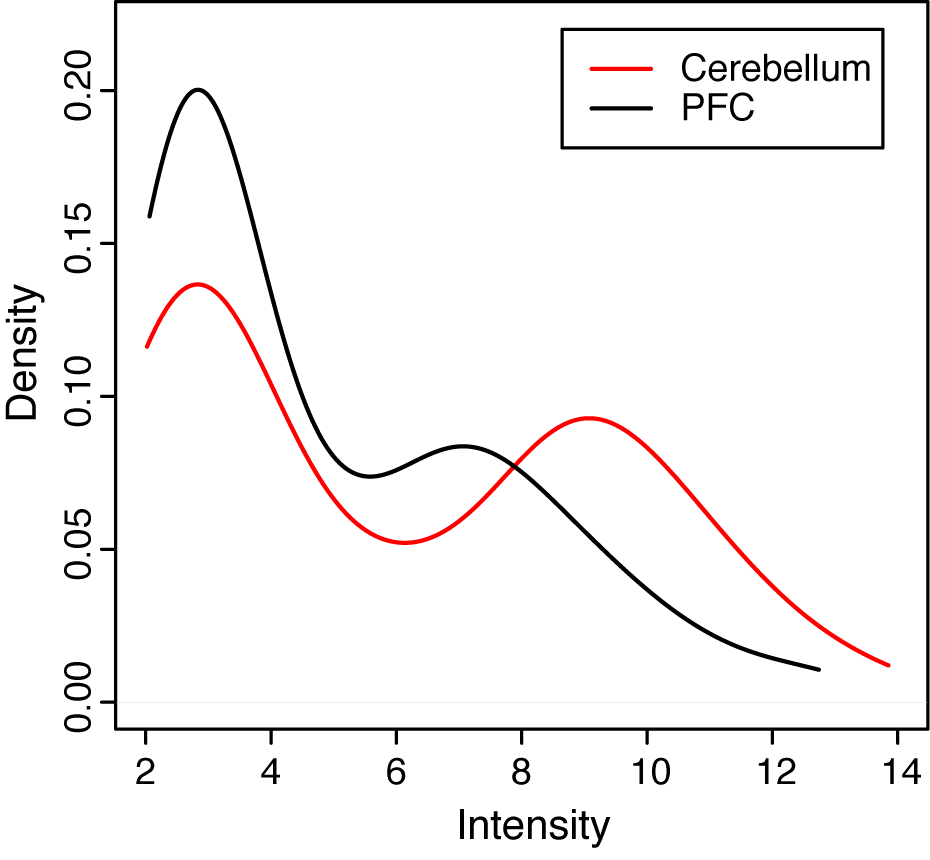

Supplement: Figure S5 — Gene expression profiles for 104 genes with CpG sites hypomethylated in cerebellum and hypermethylated in prefrontal cortex. Gene expression density distribution are plotted in log2 scale for expression intensity values (x-axis) with lines denoting cerebellum (in red) and cortex (in black). The expression data were downloaded from Gene Expression Omnibus public database (GEO accession: GSE6306). (2.42 MB TIF) [file pone.0011357.s006.tif]
