# Supplementary material for: Genome-Wide Divergence of DNA Methylation Marks in Cerebral and Cerebellar Cortices
Source: PLoS One. 2010 Jun 28;5(6):e11357. doi: 10.1371/journal.pone.0011357 (PMC2893206; doi:10.1371/journal.pone.0011357)
Supplement: Table S1 — Postmortem brain subjects used in the MSNP experiments. The color coding for subjects is consistent with those used in Figures 1, S1, S2, and S3. (0.03 MB DOC) [file pone.0011357.s007.doc]

| **Brain region** | **Subject 1** | **Subject 2** | **Subject 3** | **Subject 4** | **Subject 5** |
| --- | --- | --- | --- | --- | --- |
| Cerebellum | √ | √ | √ |  |  |
| Occipital | √ | √ | √ |  |  |
| Temporal | √ | √ | √ |  |  |
| Ventral frontal |  |  | √ | √ | √ |
